# How low can you go?

**DOI:** 10.7554/eLife.33604

**Published:** 2018-01-04

**Authors:** H Steven Wiley

**Affiliations:** Environmental Molecular Sciences LaboratoryPacific Northwest National LaboratoryRichlandUnited States

**Keywords:** EGF receptor, tumour, endocytosis, receptors, Human, Mouse

## Abstract

Extremely low numbers of active epidermal growth factor receptors are sufficient to drive tumor growth.

**Related research article** Pinilla-Macua I, Grassart A, Duvvuri U, Watkins SC, Sorkin A. 2017. EGF receptor signaling, phosphorylation, ubiquitylation and endocytosis in tumors in vivo. *eLife* **6**:e31993. doi: 10.7554/eLife.31993

Epidermal growth factor (EGF) receptors regulate a wide variety of cell behaviors, such as cell migration, proliferation, gastric acid secretion, and tissue remodeling (Chen et al., 2016). This system has been extremely well studied, yet its ubiquity across tissues and cell types has paradoxically made it difficult to understand.

A typical cell can display up to hundreds of thousands of EGF receptors on its surface. These receptors become active when ligand molecules bind to them, triggering their intrinsic kinase activity that phosphorylates both the receptor and other cellular substrates. These phosphorylation events are the signals that dictate how the cell responds to EGF. For example, adaptor molecules bind to phosphorylation sites on activated receptors, assembling molecular complexes that generate a variety of different biochemical events.

The specific biological response given out by a cell depends a lot on how many of its receptors are “occupied”, in other words, how many receptors have a ligand bound to them (Krall et al., 2011). This is likely because there is a limited number of high affinity adaptor proteins in the cell, which are needed for responses at low receptor occupancies (Shi et al., 2016; Figure 1). When a large number of the receptors are occupied, a different spectrum of signals may be produced as lower affinity adaptors engage with the receptor (Stites et al., 2015).

**Figure 1.**
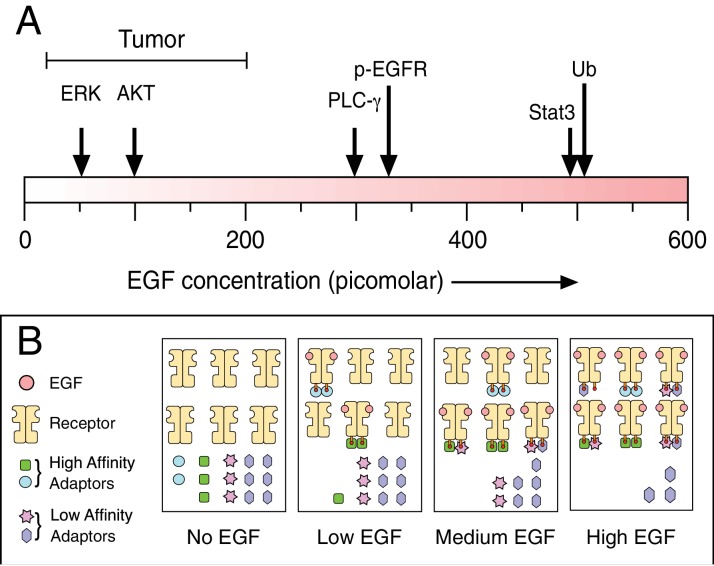
Biochemical responses to epidermal growth factor (EGF) depend on ligand dose. (**A**) As the experimental dose of EGF is increased, the spectrum of induced biochemical responses broadens. Shown are the doses required for half-maximum responses for the indicated biochemical readouts taken from Pinilla-Macua et al. ERK: Extracellular signal-regulated kinase; AKT: protein kinase B; PLC-γ: phospholipase C gamma; p-EGFR: phosphorylated EGF receptor; Stat3: signal transducer and activator of transcription 3; Ub: EGF receptor ubiquitylation. Also shown above the scale is the effective EGF dose range observed in tumors. Despite the low effective dose of EGF, this is still sufficient to drive tumor growth. One possible mechanism to explain why different biochemical responses occur at different concentrations of EGF is shown in (**B**). Following the addition of low doses of EGF, only the highest affinity adaptors can bind to the phosphorylated receptor. This drives one particular set of biochemical responses. As the high affinity adaptors are depleted at higher levels of receptor occupancy, lower affinity adaptors are then able to bind to the receptor to drive new biochemical reactions.

The EGF receptor plays a key role in many cancers, where it is often overabundant or overactive. Anti-EGF receptor therapies have proven useful in treating some cancers, but their effectiveness is limited because tumors commonly become resistant. To develop better therapies, it would be helpful to know which EGF receptor signals drive the growth of tumors. This should be predictable from the percentage of occupied receptors on the tumor cells, but this cannot be measured directly.

It should also be possible to infer the level of occupancy by measuring the amount of ligand either made by the tumor itself or available from the surrounding environment. However, locally produced ligands are consumed as fast as they are produced (DeWitt et al., 2001) and only low levels – of the magnitude of picomoles – are found in extracellular fluids accessible by tumors, such as the blood (Chien et al., 1997). This lack of information on either the level of occupied receptors or available ligands within tumors is a critical knowledge gap in identifying the signaling events that drive their growth.

Now, in eLife, Alexander Sorkin and co-workers at the University of Pittsburgh School of Medicine and Institute Pasteur – including Itziar Pinilla-Macua as first author – report how they overcame these issues (Pinilla-Macua et al., 2017). Their experimental system used a human cancer cell line, originally collected from an oral squamous carcinoma, because these cells produce up to 10 times more EGF receptors than normal cells and develop into EGF receptor-dependent tumors when grown in mice as a xenograft.

To track the receptors, Pinilla-Macua et al. used gene-editing technologies to tag the EGF receptor gene with a fluorescent protein. They then used imaging and biochemical analysis to look at how cultured cells respond when they are exposed to different doses of externally applied ligands and then compared this with what they observed in tumor xenografts.

Most of the EGF receptors in tumors were at the cell surface with only a minor fraction in early endosomes – cell compartments that normally transport the ligand-bound receptors for destruction or recycling. This corresponded with the behavior of receptors in cultured cells treated with little, if any, ligand. This is consistent with two possible explanations: low levels of receptor occupancy in tumor cells, or a fundamental difference in how the receptors behave in tumors and the cell culture system.

To decide between these two hypotheses, Pinilla-Macua et al. performed careful calibration experiments with the cultured cells to establish the relationship between ligand dose and receptor phosphorylation, a process that occurs when the receptor is activated. They then used this relationship to gauge the apparent concentration of ligand available to the tumors in vivo, yielding estimates of between 30-100 picomolar. Repeating this analysis with several other cancer cell lines and tumor xenografts yielded similar estimates. Injecting high concentrations of labeled EGF into the tumor-bearing mice increased the number of EGF receptors found in early endosomes to levels similar to that seen in cultured cells also treated with high ligand concentrations. This indicates that the receptors on tumor cells behave similarly in vitro and in vivo. Together these findings support the first of the two hypotheses: tumor cells normally have only a low level of receptor occupancy.

At these low estimated levels of occupied receptors, Pinilla-Macua et al. found that one signaling pathway that EGF receptors can trigger, called the extracellular signal-regulated kinase (ERK) pathway, reached 75-80% of its maximal activity. However, other EGF receptor-induced pathways were only marginally active. This supports previous studies that suggest that EGF receptors drive tumor growth primarily through the ERK pathway (Dhillon et al., 2007). In addition, receptor ubiquitinylation – which marks the receptors that are to be targeted to endocytosis and degradation – occurred to a similar extent in tumor cells and in cultured cells treated with picomolar concentrations of epidermal growth factor. Overall, these results make a compelling case that although just a small fraction of EGF receptors are activated in tumors, this fraction is sufficient to drive tumor growth.

So what do these results mean with respect to the more general question of biology? First, it means that the relative level of EGF receptor phosphorylation, which is directly related to occupancy, can be a poor indicator of downstream signaling. If a biological response is maximal when the receptors of a cell are well below full occupancy, then only a small degree of receptor phosphorylation will suffice. The second is that despite the large numbers of receptors found in some tumors, only a very small number need to be occupied to drive effective signaling. This is consistent with previous studies that found that the growth of tumors expressing millions of receptors is still dependent on the very small amount of ligand that they make themselves (Van de Vijver et al., 1991).

Why would some biological responses require higher concentrations of EGF than are locally available? The answer could be that they help tissues to respond to injury. High concentrations of EGF are typically only found in extracellular fluids (Zeng and Harris, 2014), which would only reach responsive cells after tissue damage. Indeed, administering high doses of EGF reduces intestinal tissue damage and improves survival in a variety of animal models (Zeng and Harris, 2014). Unfortunately, most studies on the EGF receptor have only used high doses of EGF because of the strong biochemical signals it generates. If many of the biochemical processes that drive tumor growth occur at very low levels of receptor occupancy, however, then we will need more sensitive and quantitative technologies to probe these faint molecular events.
